# Complexity: A Novel Load Progression Strategy in Strength Training

**DOI:** 10.3389/fphys.2019.00839

**Published:** 2019-07-03

**Authors:** Cauê V. La Scala Teixeira, Alexandre L. Evangelista, Paulo Eduardo de A. Pereira, Marzo E. Da Silva-Grigoletto, Danilo S. Bocalini, David G. Behm

**Affiliations:** ^1^Obesity Study Group (GEO), Federal University of São Paulo, Santos, Brazil; ^2^Department of Education, Nove de Julho University, São Paulo, Brazil; ^3^Faculty of Physical Education, Praia Grande College (FPG), Praia Grande, Brazil; ^4^Studies and Research Group of Exercise Physiology (GEPEFEX), Federal University of São Paulo, Santos, Brazil; ^5^Functional Training Group, Federal University of Sergipe, Aracajú, Brazil; ^6^Department of Physical Education, Federal University of Espírito Santo, Vitória, Brazil; ^7^School of Human Kinetics and Recreation, Memorial University of Newfoundland, St. John's, NL, Canada

**Keywords:** functional training, multicomponent training, hybrid training, resistance training, specificity

## Introduction

With physical training, the internal load is understood as the physiological responses resulting from the body's exposure to a given external load (Halson, 2014). In strength training, the external load comprises all the acute and chronic variables that can be manipulated in a session or in a training program, for example, resistance load (weight lifted), number of repetitions, speed of execution, range of motion, number of sets, rest interval between the sets and weekly frequency (American College of Sports Medicine, 2009).

Respecting the biological principles of physical training and considering the need to apply progressive overload so that training adaptations are constantly stimulated, a well-recommended approach in the scientific literature is the progression of loads (American College of Sports Medicine, 2009). This progression involves the increase or variation of the external loads, thus generating larger internal loads (muscle forces or torques) and increasing adaptations over time (Williams et al., 2017).

In the practical field and in most research involving strength training, three strategies are often explored to increase and vary the external load: (1) volume (i.e., number of sets or repetitions), (2) intensity (amount of resistive load lifted), and (3) density (i.e., alter rest periods, keeping volume and intensity unchanged). However, with the increase in the popularity of functional training (multicomponent training, task-specific training), which involves the use of strength training in a synergistic, integrated, and balanced manner with other physical capacities (La Scala Teixeira et al., 2017), and the growth of functional training in the scientific literature, has been an emergence of a more unconventional strategy of load progression: complexity.

Complexity attempts to increase the level of physical training stress/stimulus without necessarily increasing the conventional variables (i.e., load, volume, frequency). In other words, increasing the complexity increases the exercise technical difficulty, the variability in the execution patterns, and the uncertainty in the actions to be performed. Although this form of load progression has been used more frequently by coaches and researchers over the last decade, there are a limited number of studies that discuss the concept of complexity in strength training as well as the possibilities for progression that this feature offers (Suchomel et al., 2018). Thus, the aim of this technical report/opinion paper is to discuss the concept of load progression based on increasing complexity, in order to better elucidate its characteristics and make feasible its evidence-based application.

## Traditional Strategies to Increase Loads

As already mentioned, the progression of external loads as a strength training stimuli can promote continuous adaptations (American College of Sports Medicine, 2009). Furthermore, the conventional strategies often used by coaches and researchers are to increase the training volume, intensity and density.

The increase in training volume is the most common strategy as a strength training load progression, due to the ease of application, since it simply increases the resistive workload. A clear example of volume increase in training is the increase in the number of sets, repetitions, or exercises in a training session. Increased volume is a suggested strategy for load progression in subjects with related aims such as muscular hypertrophy (Schoenfeld et al., 2017; Figueiredo et al., 2018), maximal strength (Ralston et al., 2017), muscle endurance (Rhea et al., 2003b), some health parameters (Figueiredo et al., 2018), and especially in trained subjects (>1 year of training experience; Rhea et al., 2003a,b).

Increasing intensity is also a very frequent strategy, and in strength training it is to increase the external resistance (weight lifted) in the exercises (absolute intensity). Thus, increasing the intensity is a suggested strategy when the training objective is related, mainly, to the improvement of maximal strength (Rhea et al., 2003a,b; Schoenfeld et al., 2017).

The training density consists of the relationship between stimulus and recovery. In practical terms, it represents how much volume and intensity are applied over a period of time (Schoenfeld et al., 2016). The most common forms of increased training density are the reduction of rest intervals between sets and exercises (de Souza et al., 2010) and the application of advanced training techniques (e.g., drop-sets, rest-pause, circuit; La Scala Teixeira, 2016), since volume and intensity are unchanged. This strategy of load progression is generally used in training with goals related to muscular hypertrophy (de Souza et al., 2010; Prestes et al., 2017), metabolic adjustments (Paoli et al., 2012) and cardiorespiratory fitness, especially in situations in which lack of time is a limitation for the practice of physical exercise.

## Complexity: a Novel Load Progression Strategy

**Figure 1:**
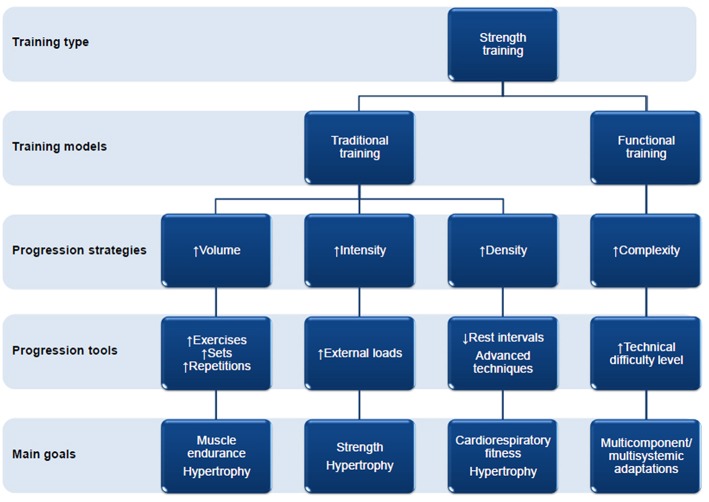
Schematic model of the progression strategies, their tools, and main goals according to the strength training model.

In the last two decades, functional training (multi-component, integrated, multi-modal, task-specific, cross training) has increased in popularity in scientific publications. Studies related to functional training have applied strength exercises (resistance exercises) with characteristics that aim to stimulate multi-systemic (or multi-component) adaptations, that is, the development of strength and other physical abilities (e.g., coordination, balance, resistance, among others) in a concomitant, integrated, and balanced manner (Lohne-Seiler et al., 2013; La Scala Teixeira et al., 2016, 2017). In this regard, it is noticed that, in the majority of the recent research that adopt models of load progression (progressive overload), although the increase of volume, intensity and/or density are present, a predominant strategy of load progression is the increase of the technical difficulty (complexity; Figure 1).

In fact, many studies comparing training protocols that included exercises with higher levels of complexity have demonstrated greater effectiveness compared to traditional programs, especially with regard to multi-systemic adaptations in children (Chaouachi et al., 2014), adults (Heinrich et al., 2012; Distefano et al., 2013), and elderly individuals (Resende Neto et al., 2016; Suzuki et al., 2018). These multi-systemic adjustments are due to the different characteristics that are explored in the strength exercises in order to raise the level of technical difficulty, consequently increasing the demand for other physical capacities.

One of the most commonly used methods to increase strength exercise complexity, especially in the elderly, is to ensure the exercise is as similar as possible to some daily life tasks (task-specific; Lohne-Seiler et al., 2013). As an example, instead of performing the bench press in its traditional form (lying on the bench), the same motor action is executed while standing, against the resistance of a cable and pulley system (e.g., crossover). Although this variation does not favor the lifting of heavy loads, which may not be optimal for maximum strength, the demand for balance, coordination, postural, and joint stability increases may favor general functional fitness (de Vreede et al., 2004, 2005; Balachandran et al., 2016). Thus, from generic exercises performed sitting or lying down, the increase of complexity can occur by performing specific, standing exercises.

Another way to increase complexity is related to the increase in execution speed, mainly in the concentric phase of the exercises. This feature, in addition to increasing neuromuscular activation (Stastny et al., 2017), increases cardiometabolic demand (Garatachea et al., 2007), and coordination (Behm and Sale, 1993), also stimulating power improvement (Fielding et al., 2002; Sayers and Gibson, 2010), with consequent improvement in the functional tasks performance (Sáez Sáez De Villarreal et al., 2010). Force-time relationships differ between training for power and maximal strength. As power is the amount of work performed over a given period of time, increasing execution speed places a greater emphasis on power outputs. Higher speed and power outputs are essential for success with most sports. Maximal strength training on the other hand, involves longer contraction durations since with the muscle force-velocity relationship, the highest forces or resistances can only be achieved at lower velocities (Behm, 1995).

The implementation of multi-segmental exercises is also used as a resource in some studies (Hoffman et al., 2004; Hedrik and Wada, 2008). Multi-segmental exercises are those that require simultaneous movement of several body segments at the same time (e.g., arms and legs) and can provide progression options for uni-segmental exercises (which mobilize only one body segment; La Scala Teixeira et al., 2017). Some examples commonly used in research and practical interventions of functional training are push press, clean high pull, and burpee. In addition to simulating daily life tasks that require simultaneous movements of the arms and legs (task-specific), multi-segmental exercises raise the level of stress on the neuromuscular and motor control systems, stimulating the concomitant development of strength, coordination, balance, mobility, and cardiorespiratory fitness (Hedrik and Wada, 2008).

Instability is another feature to increase the level of complexity in the exercises. Exercises performed on unstable bases increase the level of difficulty by disturbing the position of the body's center of gravity, which raises the demand for balance and as a consequence, joint, and core stability (Behm and Colado, 2012). Another possibility of progression of complexity is related to the use of unstable loads. For example, with similar volume (number of sets and repetitions), intensity (external load) and density (intervals between sets), exercises performed with unstable loads (e.g., dumbbells, kettlebells, elastic bands, sandbags) induce different and greater neuromuscular and coordinative demands than the same exercises performed with stable loads (e.g., machines, bars; Saeterbakken et al., 2011). Kettlebells have been shown to provide similar mechanical demand as back squats and jump exercises (Lake and Lauder, 2012). Furthermore, 6 weeks of kettlebell training improved both maximum and explosive strength and thus can provide an alternative to traditional resistance training techniques (Lake and Lauder, 2012b). Although maximal loads are decreased under unstable conditions (Behm and Colado, 2012), core (trunk) and limb muscle activation are increased when similar submaximal loads are implemented under unstable conditions (Behm et al., 2010,b). Coordination with instability changes can include decreased co-contractions and increased synergistic contributions (Behm et al., 2010,b; Behm and Colado, 2012). The Canadian Society for Exercise Physiology recommends that since unstable devices have been demonstrated to reduce low back pain incidence, as well as other functional benefits, athletes, non-athletes, and workers can incorporate unstable environments to expose themselves to a wider variety of postures and physical tasks through all planes of movement (Behm et al., 2010).

Movements performed in a multiplanar manner also present variations that contribute to the increase of training complexity. It was demonstrated that the lateral weight transfer in the squat increased the rate of perceived exertion due to the changes in coordination, balance and strength in comparison to the traditional execution (La Scala Teixeira, 2014). Moreover, when the training program included unilateral squats in multiple (three dimensional) directions there was an improvement in performance in tasks with high agility demands (Gonzalo-Skok et al., 2017).

Other resources used less frequently in studies, but not less effective in increasing complexity, are (1) the unilateral or alternating execution of exercises, which increases the coordination level, also providing changes in the activation pattern of trunk stabilizer muscles (Behm et al., 2005), (2) the execution of double task exercises, increasing the technical difficulty because the attentional focus is directed from the physical to a cognitive task (Silsupadol et al., 2009; Wollesen et al., 2017), (3) the performance of exercises with non-cyclical patterns of movement (e.g., Olympic style weightlifting), which elevates the level of coordination and improves motor control (Hedrik and Wada, 2008), and (4) exercises with visual deprivation, which increases the need for proprioception (somatosensory system) and contributes to the increase in the acute strength performance (Maior et al., 2007).

A limitation in the literature is the limited number of studies that examine or incorporate training complexity in strength training in comparison to the traditional progression variables (volume, intensity, and density). Hence, the practical application of the complexity model should consider that the literature still needs further studies whose main objective is to verify the effects, individually or jointly, of the different strategies that can be used to increase complexity in exercises/strength training. Since complex training techniques can substantially affect the loads or intensities used, the traditional variables of volume, intensity, and density must be carefully manipulated when applying complexity to inexperienced individuals.

In summary, the progression of loads by the complexity strategy seems to offer a wide range of possibilities. Several previous investigations support the use of these resources in training programs when the aim is strength enhancement simultaneously with other physical capacities in an integrated and balanced manner (multisystemic adaptations).

## Conclusion

Although volume, intensity, and density are still the most commonly used means for load progression in strength training, increased research on functional training (multi-component, integrated, multi-modal, task-specific, cross training) has suggested a new load progression strategy: complexity. Increasing the complexity involves increasing the technical difficulty level of the exercise, which raises the demand for other physical abilities during the strength exercise (coordination, balance, core stability, power, agility, among others). This strategy is suggested when the aim of the training program is to enhance muscle strength synergistically, integrated, and balanced with other physical fitness components. For this purpose, based on increasing complexity, the load progression in strength exercises is summarized below:

Generic → Specific

Lying/sitting → Standing

Uni-segmental → Multi-segmental

Uni-planar or one-dimensional → Multi-planar or three-dimensional

Slow → Fast

Stable → Unstable

Without visual deprivation → With visual deprivation

Cyclic → Acyclic

Bilateral → Unilateral

Simultaneous → Alternating

Single task → Double task.

## Author Contributions

CL, AE, PP, MD, DSB, and DGB: substantial contributions to the conception, design of the work, draft the work, and revisit it critically for important intellectual content. CL, MD, and DGB: final approval of the version to be submitted and published. CL, AE, PP, MD, DSB, and DGB: agreement to be accountable for all aspects of the work in ensuring that questions related to the accuracy and integrity of any part of work are appropriately investigated and resolved.

### Conflict of Interest Statement

The authors declare that the research was conducted in the absence of any commercial or financial relationships that could be construed as a potential conflict of interest.
